# Global Health and Visa Policy Reform to Address Dangers of Hajj during Summer Seasons

**DOI:** 10.3389/fpubh.2016.00280

**Published:** 2016-12-22

**Authors:** Mohanad Aleeban, Tim K. Mackey

**Affiliations:** ^1^Joint Masters Degree Program in Health Policy and Law, University of California San Diego School of Medicine – California Western School of Law, San Diego, CA, USA; ^2^King Fahad Medical City, Riyadh, Saudi Arabia; ^3^Department of Anesthesiology, University of California San Diego School of Medicine, San Diego, CA, USA; ^4^Division of Global Public Health, Department of Medicine, University of California San Diego School of Medicine, San Diego, CA, USA; ^5^Global Health Policy Institute, San Diego, CA, USA

**Keywords:** Hajj, Umrah, mass gatherings, religious events, pilgrimage, disaster medicine, climate change

## Abstract

Every year on the 12th month of the Islamic calendar, 2–3 million Muslims from over 160 countries migrate to Holy sites in Saudi Arabia to perform the Hajj, representing one of the largest mass gathering events worldwide. Yet, the Hajj poses several challenges to global health and public safety, including the unique health risks posed by seasonal variability when Hajj occurs during summer months. Specifically, pilgrims taking the journey to Mecca are at higher risk for heat illnesses, heat-related injuries and exhaustion, and stampedes, when summer temperatures can reach up to 48.7°C. In response, we propose that the Saudi government, in coordination with the Organization of Islamic Cooperation and the World Health Organization, explore the establishment of an expert committee, create and use a predictive risk modeling tool, and establish a dynamic quota on Hajj visas to limit potential heat exposure for high-risk populations when the Hajj falls on seasons associated with extreme weather exposure. As climate change is projected to lead to future increases in temperatures in the region, this form of dynamic and evidence-based policymaking is needed to ensure human health and safety for generations of Hajj pilgrims to come.

## Background

The Islamic religion is fast spreading throughout the world with the number of Muslims now estimated at 1.6 billion (1). Muslims have a religious obligation to participate in the Hajj once a lifetime, translating to 2–3 million pilgrims from over 180 different countries who now gather to perform the Hajj annually (2). As the worldwide population of Muslims continues to grow rapidly, the chances of attending and costs of performing the Hajj are becoming increasingly challenging. This also coincides with a Hajj that is seasonal, including months of extreme hot weather, a potential hazard that will continue for the next decade as the Hajj season is projected to take place during the summer months of the year (June–September).

As one of the world’s largest mass movement and gathering of people, the Hajj has been associated with several health concerns including communicable disease spread, increased burden of chronic disease, environmental health concerns, trauma and injury, and security risks (2–7). Further risks arise from the seasonal variability of the event, which introduces its own unique health-related challenges, including the need to engage in additional protective behaviors in response to environmental exposure, pollution, and even the effects of climate change (4, 8).

Hajj seasonal variability is driven by when it is held: every year in the 12th month of the Islamic (lunar) calendar (from the 8th to 13th of Dhu al-HIjja) (3). However, the lunar calendar only has 354 days, compared with the 365 days for Gregorian calendar, which is used as the international standard. This means that the Hajj is held during different periods of seasonality generally every 9 years.

## Health Risks Associated with Seasonal Variability

Years when the Hajj falls under the summer season can lead to higher risk of heat illnesses (e.g., heatstroke, heat injuries and exhaustion, heat hyperpyrexia, skin milia, and dehydration) and stampedes, where the temperature reaches between 43 and 48.7°C, and has a relative humidity of 58–87%. Statistics from the Saudi Ministry of Health indicate that there is a close relationship between heat casualties and climatic heat load during Hajj seasons (3, 4). Heat illness can occur due to high temperatures, strenuous physical exertion during the performance of rituals, and overcrowding, particularly when there are extreme temperatures (i.e., temperatures as high as 45°C) (9). For the Hajj that took place in September 2015, 1,737 heat illness cases were recorded, with 52.2% occurring among pilgrims between the ages of 51 and 70 years (10). The increasing risk of heat exposure is becoming more apparent, given that there were only 796 total cases that occurred over the preceeding 10 Hajj seasons (10, 11).

In addition, multiple deadly stampede incidents (around eight incidents) have occurred in the last three decades, with a primary cause for these public safety events related to crowd flow dynamics. Fatalities have ranged between 14 and 346 deaths (4), though two major stampede incidents in 1990 and 2015 tragically resulted in a much higher number of fatalities (3, 12). In the summer of 1990, more than 1,400 pilgrims died from a stampede inside a pedestrian tunnel in Mina after a cooling system failure (3). In summer 2015, one of the world’s most deadliest stampedes occurred at a small intersection again in Mina, resulting in conflicting reports of fatalities with some estimates as high as 2,000 deaths (12). Though results from an official investigation have yet to be disclosed, many suggest that the limited space and crowd flow dynamics were major contributors for the incidents. Notably, all incidents occurred in Mina Valley (that has a maximum capacity of only 1.4 million).

Importantly, both the 1990 and 2015 incidents shared common risk characteristics; both had exceedingly high casualties compared to the other stampedes and both occurred during the summer seasons (July to September) of Hajj. Although excessive heat may not be the primary cause of stampede events, it has arguably played a critical role in contributing to a higher number of casualties given that stampede events in summer months have had higher mortality compared to stampedes that have occurred in other seasons.

## Hajj Visas

As the Hajj continues to grow as a major global event, it is critical that public policies addressing the Hajj take into account the need to dynamically protect the public health and the safety of pilgrims in the face of uncertainty with rising temperatures and global climate change and also plans to increase the number of pilgrims that will receive Hajj visas (8). This includes proactively assessing the potential risks of heat exposure that can lead to poor health outcomes and the role of high temperatures and overcrowding that can exaggerate the number of casualties when stampedes inevitably occur. Importantly, these risks are likely to accentuate, with the Hajj set to take place during the summer seasons for the next decade. Specifically, we believe that health risks associated with summer Hajj can be mitigated by adjusting how the Saudi government allocates “Hajj” and “Umrah” (minor pilgrimage) visas, while also creating financing mechanisms to ensure better equitable participation for all pilgrims to perform the Hajj.

Specifically, Hajj and Umrah visas have similar requirements but differ in the time of year they are issued and their duration (see Table 1). The Hajj visa is specific to the Hajj season, with foreigners (e.g., non-Saudi Arabian nationals/residents) required to obtain visas by applying to the Saudi embassy in their respective country. In comparison, for Umrah visas, Muslims can visit areas of the Holy site that are open outside the Hajj season, limited to 15 days in duration. In addition to visa requirements for foreigners to travel and perform the Hajj, there is also a permit requirement for Saudi residents (both citizens and non-citizens).

**Table 1:** **Comparison between Hajj and Umrah**.

	Hajj	Umrah
Islamic obligation	Conditional obligation	Less obligatory than the Hajj
Duration of the rituals	5–6 days	As short as a few hours
Time	Specific time period (12th month of Islamic calendar)	Open all year except during the Hajj season
Place(s) of ritual	Multiple places: Mecca, Arafat, Mina, and Muzdalifah. All pilgrims obligated to spend certain nights at specific sites	Grand Mosque in Mecca, without any obligation to spend time at specific sites
Visa duration	Open during Hajj season only (approximately 2-month duration)	Open all year around except Hajj season (with 30 days validity and 15-day limit of stay for each pilgrim)
Visa quota	1 pilgrim per 1,000 residents for each Islamic country annually	Variable allocation
Approximate number of expected pilgrims	2–3 million with plans to increase to 5 million by 2030	More than 5 million in 2015, with plans to increase to 30 million by 2030

Prior to 2008, residents of Saudi Arabia could perform Hajj and Umrah without a permit or designated lodging. However, since 2008, residents must obtain a Hajj permit through the Ministry of Interior to gain entry to the holy areas, though no permit is required to perform Umrah (13). The general requirements for a Hajj permit for both Saudi citizens and non-citizen residents include that they must have not performed the Hajj within the past 5 years and that they contract with an approved tour company (i.e., “Hamlah”) to provide transportation and lodging. They must also satisfy vaccination requirements updated annually by the Saudi Ministry of Health (14). Additionally, non-citizens are required to produce additional documentation (e.g., proof of legal status, no-objection letter from the employer) (15). Violating Hajj permit laws may result in paying a fine, incarceration or immediate deportation, and a ban from entering the country in the future (for non-citizens) (16).

## Policy Proposal

Given the well-documented public health challenges associated with the Hajj during summer months, we propose that the Saudi government, in coordination with the Organization of Islamic Countries (OIC) and in partnership with the World Health Organization and its Regional Office for the Eastern Mediterranean, create a permanent expert committee to proactively assess Hajj-related health risks related to climate. The committee should also lead in the development and use of a predictive risk modeling tool, used prior to the start of Hajj, which would assess the unique climate-related health risks associated with the annual pilgrimage and provide guidance on identifying pilgrims who are at the highest risk of adverse heat exposure.

The risk modeling tool [we note that predictive modeling has been used for several health challenges including in the acute care setting, for population health, mental health, to model infectious disease risk, and for assessing the potential impact of climate change on health (17–21)] could generate evidence for use in national surveillance systems and inform the committee’s guidance and technical assistance process. This process would specifically focus on proactively assessing existing and emerging health risks associated with extreme temperatures during the Hajj that are likely to be impacted by climate change. It could also provide the evidentiary basis for adjustments to the number of Hajj visas issued each year, designed to ensure the safety of at-risk pilgrims and avoid potential overcrowding that could lead to additional public safety concerns.

The visa adjustment process could be accomplished by establishing a quota system on the allocation of Hajj visas issued for pilgrims who are already recognized as most vulnerable to the risks of heat illness (including the elderly, those with pre-existing illnesses, and children accompanying parents) (3, 4, 22). Importantly, the quota would aim to limit the percentage of the vulnerable population that could be exposed, rather than the aggregate number of pilgrims issued visas, and would only be triggered when the Hajj occurs during summer months and other seasons with extreme weather or climate events. Screening to determine if pilgrims fall under the “at-risk” criteria could occur through the existing visa and permitting process and associated health screenings. For years when the quota is instituted, the Saudi government would set aside additional visas for excluded populations when Hajj occurs during months that are cooler and could also compensate pilgrims by adding additional numbers of Umrah visas in the year of exclusion.

Importantly, we recognize that the Hajj is a far greater obligation in the Islamic faith compared to Umrah. However, the dream of performing the Hajj, or even visiting the Holy sites, can become unachievable for many Muslims, due to multiple factors including the increasing number of Muslims, Holy site capacity restrictions, and the high cost of the Hajj. The solution of simply increasing the visa quota will likely not address this enormous demand and the various barriers to participation, yet may cause a significant increase in the health risks associated with the event. Hence, in order to ensure the safety of pilgrims at the Hajj and enhance equitable access, the Saudi government and OIC should dynamically assess the use of quotas to *both* mitigate potential health risks and concomitantly to reallocate visas to ensure more equitable access.

A policy proposal to adjust the number of visas available for the Hajj is not unprecedented. In 1987, after consultations with other Islamic countries at the OIC, the Saudi government reached a decision to set a Hajj visa quota (1 pilgrim per 1,000 residents for each Muslim country.) In 2013, the Hajj quota was reduced by 20 and 50% for international and domestic pilgrims, respectively, in response to planned construction in the Holy areas designed to accommodate larger numbers of future pilgrims (23). Visa adjustments should also be done in combination with other public health interventions (e.g., establishment of cooling stations, shaded structures, awareness campaigns that emphasize the importance of proper hydration, and use of sun protection measures) to ensure all pilgrims are protected from heat and other climate-related exposure (24).

Parallel to the proposed quota system, the OIC should also explore a multilateral financing mechanism to help as many Muslims as possible to gain access to the resources they need to perform the Hajj earlier in their lifetime and when they are healthy enough to do so. The financing mechanism should be focused on providing subsidies to those pilgrims who cannot normally afford the pilgrimage and also encouraging equitable participation by prioritizing those who have not previously preformed the Hajj. Additionally, as Hajj obligations are only mandatory for those pilgrims who are “physically and financially” able, additional Umrah visas will also provide pilgrims other opportunities to visit the Holy site in Mecca outside of the summer season (25).

## Discussion

The policy proposals we suggest here are in conjunction with others who have advocated for the use of health screenings to potentially exclude the participation of seriously ill pilgrims (25). We argue that the documented health hazards occurring during summer Hajj provide justification for evidence-based policy reform of the Hajj assessment and visa allocation process. If the Hajj quota can be reduced to accommodate planned infrastructure improvements, there is good reason to begin an international dialogue on the potential utility of forming an expert committee to specifically assess climate and the Hajj and also to explore the use of seasonal visa quotas aimed at accommodating adjustments to protect the most vulnerable pilgrims from potentially fatal heat exposure. Further, as infrastructure improves to protect pilgrims from dangerous heat exposure (e.g., air conditioned tents, tunnels, access to health-care facilities, canopies, and mist sprinklers) and accommodate more capacity, the quota’s percentage can be adjusted/increased accordingly.

As climate change is projected to make summertime conditions at the Hajj a higher risk to human health and mass migration now and into the future, the international community, lead by the Saudi government, OIC, and broader Islamic community, should actively pursue adaptive policymaking that enhances Hajj visa allocation with the aim of promoting public safety and global health.

## Author Contributions

MA and TM jointly conceived the study, wrote the manuscript, and edited the final manuscript.

## Conflict of Interest Statement

The authors declare that the research was conducted in the absence of any commercial or financial relationships that could be construed as a potential conflict of interest.
